# EEG resting-state functional connectivity: evidence for an imbalance of external/internal information integration in autism

**DOI:** 10.1186/s11689-022-09456-8

**Published:** 2022-08-27

**Authors:** Wantzen Prany, Clochon Patrice, Doidy Franck, Wallois Fabrice, Mahmoudzadeh Mahdi, Desaunay Pierre, Mille Christian, Guilé Jean-Marc, Guénolé Fabian, Eustache Francis, Baleyte Jean-Marc, Guillery-Girard Bérengère

**Affiliations:** 1 Normandie univ, UNICAEN, PSL Université Paris, EPHE, INSERM, U1077, CHU de Caen, GIP Cyceron, Neuropsychologie et Imagerie de la Mémoire Humaine, 14000 Caen, France; 2 Université de Paris, LaPsyDÉ, CNRS, F-75005 Paris, France; 3 INSERM UMR-S 1105, GRAMFC, Université de Picardie-Jules Verne, CHU Sud, 80025 Amiens, France; 4 Centre Ressources Autisme Picardie, Service de Psychopathologie Enfants et Adolescents, CHU, 4 rue Grenier et Bernard, 80000 Amiens, France; 5 Service de Psychiatrie de l’enfant et de l’adolescent, Centre Hospitalier Interuniversitaire de Créteil, 94000 Créteil, France

**Keywords:** Autism spectrum disorder, EEG, Alpha, DMN, DAN, SMN, Connectivity, Integration, Resting state

## Abstract

**Background:**

Autism spectrum disorder (ASD) is associated with atypical neural activity in resting state. Most of the studies have focused on abnormalities in alpha frequency as a marker of ASD dysfunctions. However, few have explored alpha synchronization within a specific interest in resting-state networks, namely the default mode network (DMN), the sensorimotor network (SMN), and the dorsal attention network (DAN). These functional connectivity analyses provide relevant insight into the neurophysiological correlates of multimodal integration in ASD.

**Methods:**

Using high temporal resolution EEG, the present study investigates the functional connectivity in the alpha band within and between the DMN, SMN, and the DAN. We examined eyes-closed EEG alpha lagged phase synchronization, using standardized low-resolution brain electromagnetic tomography (sLORETA) in 29 participants with ASD and 38 developing (TD) controls (age, sex, and IQ matched).

**Results:**

We observed reduced functional connectivity in the ASD group relative to TD controls, within and between the DMN, the SMN, and the DAN. We identified three hubs of dysconnectivity in ASD: the posterior cingulate cortex, the precuneus, and the medial frontal gyrus. These three regions also presented decreased current source density in the alpha band.

**Conclusion:**

These results shed light on possible multimodal integration impairments affecting the communication between bottom-up and top-down information. The observed hypoconnectivity between the DMN, SMN, and DAN could also be related to difficulties in switching between externally oriented attention and internally oriented thoughts.

**Supplementary Information:**

The online version contains supplementary material available at 10.1186/s11689-022-09456-8.

## Background

Autism spectrum disorder (ASD) refers to neurodevelopmental disorders characterized by social communication difficulties and restricted and repetitive behaviors [1]. Studies using functional magnetic resonance imaging (fMRI) and electroencephalography (EEG) at rest have consistently found atypical functional connectivity in ASD that goes some way to explaining its symptoms (see [2–4], for a review). Importantly, it has been proposed that during the so-called resting-state (RS) periods, the brain integrates and processes information in an active way [5]. Thus, atypical functional connectivity at rest in ASD may indicate disintegration of the information through the brain.

In this context, alpha oscillation appears as an interesting marker of dysfunctions [6]. Alpha oscillation (7.5–12.5 Hz) is the main rhythm during unconstrained brain activity (i.e., resting state), and it decreases when subjects are asked to perform a task [7]. These alpha rhythms seem to act as a traffic controller of information flow within the cortex and are thought to be an index of cortical inhibition [7–9] via top-down inhibitory control through modulation of the neural excitation/inhibition balance [7]. Alpha oscillation is inversely related to externally oriented attention, reflecting functional inhibition of sensory systems [10]. Alpha suppression has been consistently linked to increased attention and vigilance [11, 12].

In ASD, most resting-state studies have reported decreased connectivity in the alpha band in ASD [13–22] but some observed increased connectivity [23–25]. Many EEG studies in ASD include a large range of ages with few participants: 15 ASD participants (9–18 years, [26]); 9 ASD participants (12–53 years, [27]); 10 ASD participants (21–41 years, [28]); 10 ASD participants (16–38 years, [29]); and 19 ASD participants (7–17 years, [30]). Studies with larger sample sizes are still needed. These mixed results concerning alpha connectivity are difficult to compare due to the use of very different methods (acquisition method, condition of rest, recording durations, region of interest, connectivity metric, etc.) However, considering the literature, it is still possible to envisage a decrease in these electrophysiological indexes in most people with autism.

Mathewson and colleagues [31] showed an association between reduced coherence in posterior regions with preferential attention to detail in ASD groups (as measured by the autism spectrum quotient (AQ)). In this vein, impaired neural synchrony may be a primary pathophysiological mechanism in ASD, contributing to atypical functional connectivity [32, 33], and could represent an endophenotype that underlies the information processing impairments in ASD [34, 35].

Most of the connectivity findings in EEG have been based on measurements between pairs of electrodes, which have several limitations, including volume conduction [36] and poor estimation of anatomical locations (presumption of a 2- and not 3-dimensional space). 3D analyses of EEG sources and connectivity may provide more detailed and accurate information. However, most studies in ASD have focused on EEG connectivity investigations on scalp measurements. One single case study in ASD, using correlation analyses between current source density measure (implemented by standardized low-resolution brain electromagnetic tomography (sLORETA)), showed increased short connectivity, between regions involved in the mirror neuron system and social perceptual networks [37]. One MEG study used source localization approach and focused on RS networks (RSNs) in ASD, including the default mode network (DMN) and the salience network [38]. The authors showed lower gamma-band connectivity within the DMN and between the DMN and salience network, which correlated with the severity of ASD symptomatology in social communication and interaction abilities.

The DMN is involved in top-down processes related to internal-oriented thought abilities, such as self-reflection, mental representation, perspective-taking, and autobiographical memory [39, 40]. Altered activation and connectivity within the DMN in ASD have emerged as a key system underlying social dysfunction (see [41] for review).

Furthermore, while a growing interest over the last decade concerns atypical sensory processing — a new diagnostic criterion of ASD includes in the DSM-5 [1] — few studies have focused on the sensorimotor network (SMN). Atypical sensorimotor and perceptual processes suggest an impaired SMN [42–44] that is linked to the core symptoms of ASD [45–48].

In the same way, although attentional atypicality is well known in ASD, very few studies have explored brain functional networks underpinning attentional processes. The dorsal attention network (DAN) has been linked to the regulation of goal-directed top-down processing [49]. Dysconnectivity in this network may explain attentional process difficulties in ASD [50, 51].

Most RS studies in ASD have been conducted using fMRI, and few have focused on the previously mentioned RSN, specially. Exploring the connectivity of the alpha band is of particular interest as it is related to the coordination of distant brain regions [7, 52, 53]. The alpha band is positively related to the DMN and the somatosensory system [54–56] and negatively correlated with the DAN [53]. Thus, these approaches offer new insights into the mechanisms underlying the atypical functional organization of brain networks in ASD (e.g., high temporal resolution, specific oscillatory frequency-band effects, a direct measure of brain activity) that fMRI cannot reveal [57, 58]. Thus, we focus on SMN, DMN, and DAN which here we refer as 3-RSNs.

Atypicality has been reported within these 3-RSNs, but data are very sparse concerning connectivity between them in ASD [38, 59, 60]. Between-network connectivity reflects the amount of information integration between different networks, which is crucial for many functions impacted in ASD, such as perception, social interaction, and communication [61, 62].

The present study aimed to provide a detailed description of the activity and connectivity profiles within and between the 3-RSN through alpha rhythms in ASD compared to typically developing (TD) participants. We expected to observe underconnectivity both within and between the SMN, the DAN, and the DMN in the ASD group, indicating poor brain communication. This reduced functional connectivity may reflect the existence of a core multimodal integration deficit affecting the communication between bottom-up and top-down information.

## Methods

### Participants

We recruited 29 participants with ASD and 38 TD controls (mean 16.1 and 16.5 years respectively, Table 1) via two autism resource centers in France. All participants were right-handed and matched for sex, age, and intellectual quotient (Table 1). This study is interested in major brain functioning abnormalities found in ASD during adolescence. We have used Sawyer et al. (2018)’s publication [63] as a reference for defining the period of adolescence, including participants aged from 10 to 25 years old. As mentioned above, there are studies covering a wide age range but none on this period specifically and with a relatively large number of participants. Furthermore, to keep a homogenous age range between groups, we matched ASD individuals and controls on a case level by age.

**Table 1:** Population description

	ASD (n = 29)			TD (n = 38)			Group comparison
	Mean	SD	Range	Mean	SD	Range	
**Age (years)**	16.1	3.6	11.0–25.7	16.5	4.2	10.2–25.6	0.693
**FSIQ**	99.1	14.0	72–128	104.8	10.2	86–126	0.060
**AQ total**^a^	34.3	9.4	11–48	11.4	5.0	2–22	<.0001

Mean, standard deviation (*SD*), range, and analyses for group differences (Student’s *t*-test) for age, Full-Scale Intelligence Quotient (FSIQ), and Autistic Quotient (AQ)

^a^28 ASD participants completed the AQ

These participants with ASD had received a clinical diagnosis of verbally and intellectually high-functioning autism according to DSM-5 criteria [1]. All ASD participants were diagnosed by the Autism Diagnostic Interview-Revised [64] (17 ASD participants) and/or the Autism Diagnostic Observation Schedule-Generic (ADOS, [65]) (15 ASD participants). All ASD and TD participants have completed the AQ [66] except for one ASD participant. For all participants, exclusion criteria were a history of neurological disorders or psychiatric illness (other than ASD in the ASD group), a first-degree relative with ASD in the TD group, head trauma, medication interfering with the EEG signal, intellectual disability, and learning disabilities. All participants voluntarily took part in the study, and written consent was obtained from children and their parents after providing them with detailed information. This research was undertaken following the Declaration of Helsinki and approved by the regional ethics committee (CPP Nord Ouest III). It was supported by the French Ministry of Health (PHRC, ID-RCB: 2014-A00481-46). Each participant underwent a Wechsler Intelligence Scale: the Wechsler Intelligence Scale for Children-Fourth Edition [67] for children aged 6–17 years and the Wechsler Adult Intelligence Scale-Fourth Edition [68] for participants aged 18 years or above.

### EEG recording and data acquisition

Continuous EEG recording and data acquisition procedures were similar across research centers. They were performed in a faraday cage for 3 min in an awake RS eyes-closed condition, using an EGI Hydrocel Geodesic Sensor Net (HGSN-130) dense array of 128 Ag/AgCl sensors [69] (Electrical Geodesics Inc., Eugene, OR, USA). Considering our hypothesis and interest in the alpha band, we chose to study the eyes-closed condition. Alpha waves are mainly found with eyes closed and decrease with eyes opened [7, 70, 71]. Moreover, an eyes-closed condition would provide a closer measure of resting activity than open eyes because it minimizes cognitive load, goal-directed action, and external stimulation [72, 73]. Participants were instructed to relax, remain as still as possible, and close their eyes. Impedances were kept under 100 kΩ [74], and the EEG channel was referenced to a vertex reference (fixed by the EGI system). EEG data were processed offline using Netstation 4.4.2 (Electrical Geodesics Inc., Eugene, OR, USA) before they are being exported (see the detailed method in [59]). The signal was sampled at 1 kHz and filtered using a low-pass filter at 500 Hz and a high-pass filter at 1 Hz. After processing, nonoverlapping 2.048-s epochs were extracted for analysis. The EEG was visually screened by two experts (the first authors, PW and PC). The remaining artifacts (e.g., saccades, muscle contractions, head movement, etc.) were excluded manually for the analysis epoch by epoch before re-referenced to a common average reference. All participants had a minimum of 60 s of artifact-free data available for analysis (30 epochs of 2.048 s minimum per subject, ASD: mean number of epochs: 40.7, SD 11.5, range 30–69; TD: mean number of epochs: 44.1, SD 10.9, range 30–66), respecting Pascual-Marqui et al. (2011)’s guidelines [75].

We focused our spectral analysis on the alpha frequency band (7.5–12.5 Hz, [16, 76–78]) given our hypothesis.

### Source localization analysis and statistical non-parametric mapping (SnPM)

Using sLORETA, we estimated the current density recordings of source signals in a standardized brain atlas space, utilizing a restricted inverse solution (for details, see [79, 80]). Current source density distributions in the ASD and TD groups were compared in a voxel-by-voxel analysis of sLORETA data for the alpha frequency band. We submitted the sLORETA images to statistical non-parametric mapping (SnPM) for each contrast, applying a *t* statistic to log-transformed data for unpaired groups. We log-transformed sLORETA images before the statistical analyses for each participant to reduce confounds with no regional specificity [81]. Correction for multiple comparisons was applied in SnPM with random permutations (5000 in the current study) and has been shown to yield results similar to those obtained from statistical parametric mapping with a general linear model and multiple comparison corrections derived from random field theory [82, 83]. *A t*-threshold (*t* = 3.426) corresponding to a statistical significance threshold (*p* < 0.05) was calculated using the statistical tool provided by sLORETA. Only regions with at least five significant voxels were retained.

### Functional connectivity analysis

#### Regions of interest

To evaluate between-group connectivity differences, we conducted analyses with different the 3-RSNs. We selected seeds from key regions within the SMN, DMN, and DAN, from the parcellations described by Wu et al. [84] and Yeo et al. [85], resulting in five regions of interest (ROIs) for the SMN, four for the DMN, and twelve for the DAN. SMN coordinates were transformed from Talairach to MNI coordinates.

ROIs were created by including all gray-matter voxels within a 10-mm radius of the seed. The log-transformed electric current density was averaged across all voxels belonging to a given ROI. LORETA gave the names of all the regions to the corresponding coordinates (listed in Table 2). For DAN, two voxels were each labeled *precuneus right* by LORETA nomenclature. As one of them was very close to the right superior parietal, we labeled it *precuneus-superior parietal lobule R* to differentiate it from the second voxel located more in the center of the precuneus.

**Table 2:** Cortical regions of interest for RSN functional connectivity

RSNs	Regions	MNI coordinates		
		***x***	***y***	***z***
**SMN**	**Precentral gyrus R**	34	−32	59
	**Postcentral gyrus L**	−44	−30	58
	**Medial frontal gyrus**	−4	−33	66
	**Transverse temporal gyrus L**	−56	−23	12
	**Transverse temporal gyrus R**	55	−23	12
**DAN**	**Middle frontal gyrus R**	22	−8	54
	**Middle frontal gyrus L**	−22	−8	54
	**Inferior parietal lobule R**	34	−38	44
	**Inferior parietal lobule L**	−34	−38	44
	**Precuneus R**	18	−69	51
	**Precuneus L**	−18	−69	51
	**Middle temporal gyrus R**	51	−64	−2
	**Middle temporal gyrus L**	−51	−64	−2
	**Precuneus-superior parietal lobule R**	8	−63	57
	**Superior parietal lobule L**	−8	−63	57
	**Inferior frontal gyrus R**	49	3	34
	**Inferior frontal gyrus L**	−49	3	34
**DMN**	**Superior temporal gyrus R**	41	−60	29
	**Superior temporal gyrus L**	−41	−60	29
	**Medial frontal gyrus**	0	49	18
	**Posterior cingulate cortex**	0	−52	26

*SMN* sensorimotor network, *DAN* dorsal attention network, *DMN* default mode network, *L* left, *R* right

#### Functional connectivity analyses

The connectivity analyses were performed by computing lagged phase synchronization using sLORETA. Lagged phase synchronization provided the similarity (a corrected phase synchrony value) between signals in the frequency domain based on normalized Fourier transforms. This measure corrects the volume conduction effects, intrinsic physics artifacts or non-physiological effects, and low spatial resolution. It represents a non-linear connectivity measure between two regions after excluding this zero-lag contribution (for more details on the sLORETA connectivity algorithm, see [75]). In this respect, it is considered to contain only physiological connectivity [57].

We examined group differences on within- and between-network connectivity by comparing lagged phase synchronization between ROIs for each artifact-free EEG segment in the alpha frequency band. Analyses were conducted using a one-tailed *t* statistic on log-transformed data corrected for multiple comparisons with a non-parametric permutation procedure (5000 randomizations). The *t* thresholds corresponding to statistical significance thresholds (*p* < 0.05) were calculated for connectivity analyses within the SMN (*t = 2.604*), DAN (*t =* 3.127), and DMN (*t =* 2.412) and for differences in connectivity between the SMN and DAN (*t =* 3,343), SMN and DMN (*t =* 3.019), and DAN and DMN (*t =* 3.291).

LORETA using non-parametric permutation tests, we pursued analyses by extracting connectivity values (within- and between-RSN functional connectivity) that were significantly different between groups to conduct backward stepwise regression analyses with age and ASD symptomatology (AQ) as individual predictors.

## Results

### Source localization

**Fig. 1:**
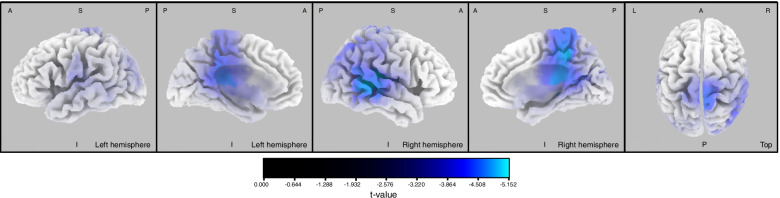
Source localization differences between ASD and TD groups in the alpha frequency band. Blue areas represent the spatial extent of voxels with a significantly reduced current source density between ASD and TD groups (*p* < 0.05 corrected for multiple testing, *t* < −3.426). L left, R right, A anterior, P posterior, S superior, I inferior

In the ASD group, relative to the TD group, source localization analyses in the alpha band showed significantly decreased current source density. Reduced current source density was localized in temporoparietal and somatosensory/medial areas, in the main regions involved in the DMN, the SMN, and the DAN (Fig. 1, see Additional file 1). No difference was found for ASD > TD.

### Functional connectivity within RSNs

**Fig. 2:**
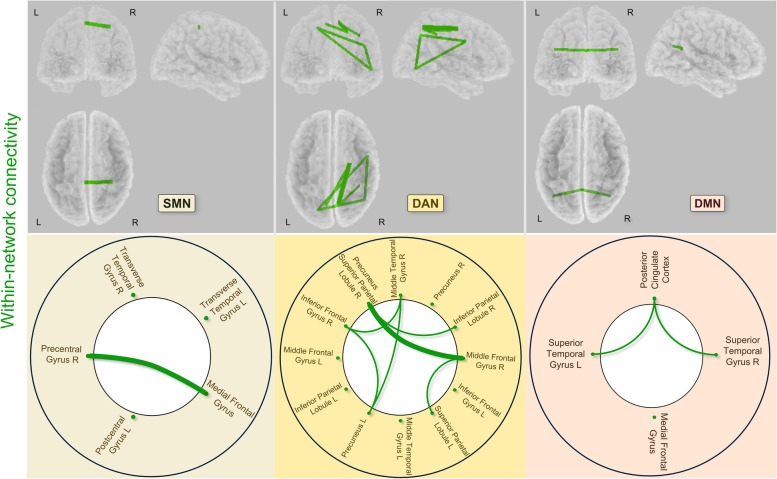
Connectogram of significant differences in within-RSN connectivity in the ASD group relative to the TD group. Lines indicate significantly decreased lagged phase synchronization for the ASD group relative to the TD group within SMN, DAN, and DMN (thin line *p* < 0.05, thick line *p* < 0.01). L left, R right

We performed statistical analyses with sLORETA software to assess between-group differences in lagged phase synchronization within the 3-RSNs, namely the SMN, DAN, and DMN (Table 3, Fig. 2 and summary Fig. 4). No difference was found for ASD > TD. Within the SMN, we identified significantly reduced connectivity between the medial frontal gyrus and right precentral gyrus in the ASD group relative to the TD group (*t* = −3.555, *p* < 0.005). Within the DAN, statistical analyses revealed reduced connectivity in the ASD group relative to the TD group (1) between the right precuneus-superior parietal lobule and both the right middle frontal gyrus (*t* = −3.817, *p* < 0.009) and the right inferior parietal lobule (*t* = −3.247, *p* < 0.038), (2) between the left precuneus and both the right middle temporal gyrus (*t* = −3.764, *p* < 0.011) and the right inferior frontal gyrus (*t* = −3.503, *p* < 0.020), (3) between the right middle temporal gyrus and the right inferior frontal gyrus (*t* = −3.315, *p* < 0.032), and (4) between the right middle frontal gyrus and the left superior parietal lobule (*t* = −3.160, *p* < 0.046). Within the DMN, we identified significantly decreased connectivity between the posterior cingulate cortex and the right and left superior temporal gyrus (*t* = −2.559, *p* < 0.038, *t* = −2.892, *p* < 0.018, respectively) in the ASD group relative to the TD group.

**Table 3:** Significant differences in within-RSN functional connectivity in the ASD group relative to the TD group

Regions	MNI coordinates			Regions	MNI coordinates			*t*	*p*
	*x*	*y*	*z*		*x*	*y*	*z*		
SMN									
Medial frontal gyrus	−4	−33	66	Precentral gyrus R	34	−32	59	−3.555	<0.005
DAN									
Precuneus-superior parietal lobule R	8	−63	57	Middle frontal gyrus R	22	−8	54	−3.817	<0.009
Precuneus L	−18	−69	51	Middle temporal gyrus R	51	−64	−2	−3.764	<0.011
Precuneus L	−18	−69	51	Inferior frontal gyrus R	49	3	34	−3.503	<0.020
Middle temporal gyrus R	51	−64	−2	Inferior frontal gyrus R	49	3	34	−3.315	<0.032
Precuneus-superior parietal lobule R	8	−63	57	Inferior parietal lobule R	34	−38	44	−3.247	<0.038
Middle frontal gyrus R	22	−8	54	Superior parietal lobule L	−8	−63	57	−3.160	<0.046
DMN									
Posterior cingulate cortex	0	−52	26	Superior temporal gyrus L	41	−60	29	−2.892	<0.018
Posterior cingulate cortex	0	−52	26	Superior temporal gyrus R	−41	−60	29	−2.559	<0.038

*SMN* sensorimotor network, *DAN* dorsal attention network, *DMN* default mode network, *L* left, *R* right

### Functional connectivity between RSNs

**Fig. 3:**
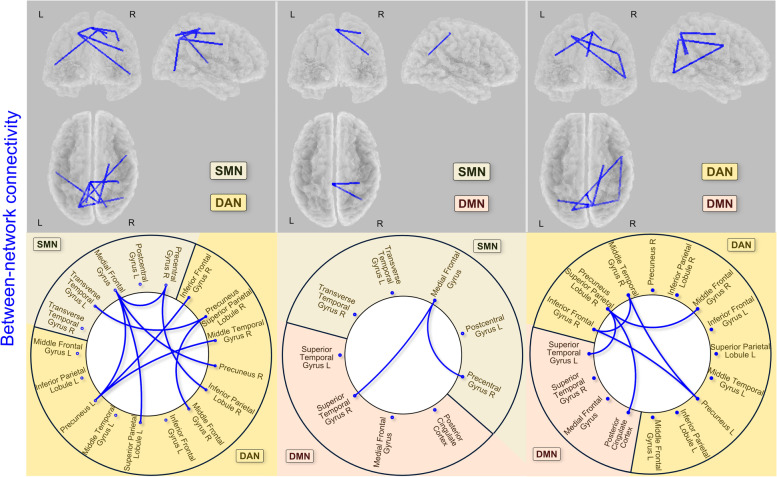
Connectogram of significant differences in between-RSN connectivity in the ASD group relative to the TD group. Lines indicate significantly decreased lagged phase synchronization for the ASD group relative to the TD group between SMN and DAN, between SMN and DMN, and between DAN and DMN (thin line *p* < 0.05). L left, R right

**Fig. 4:**
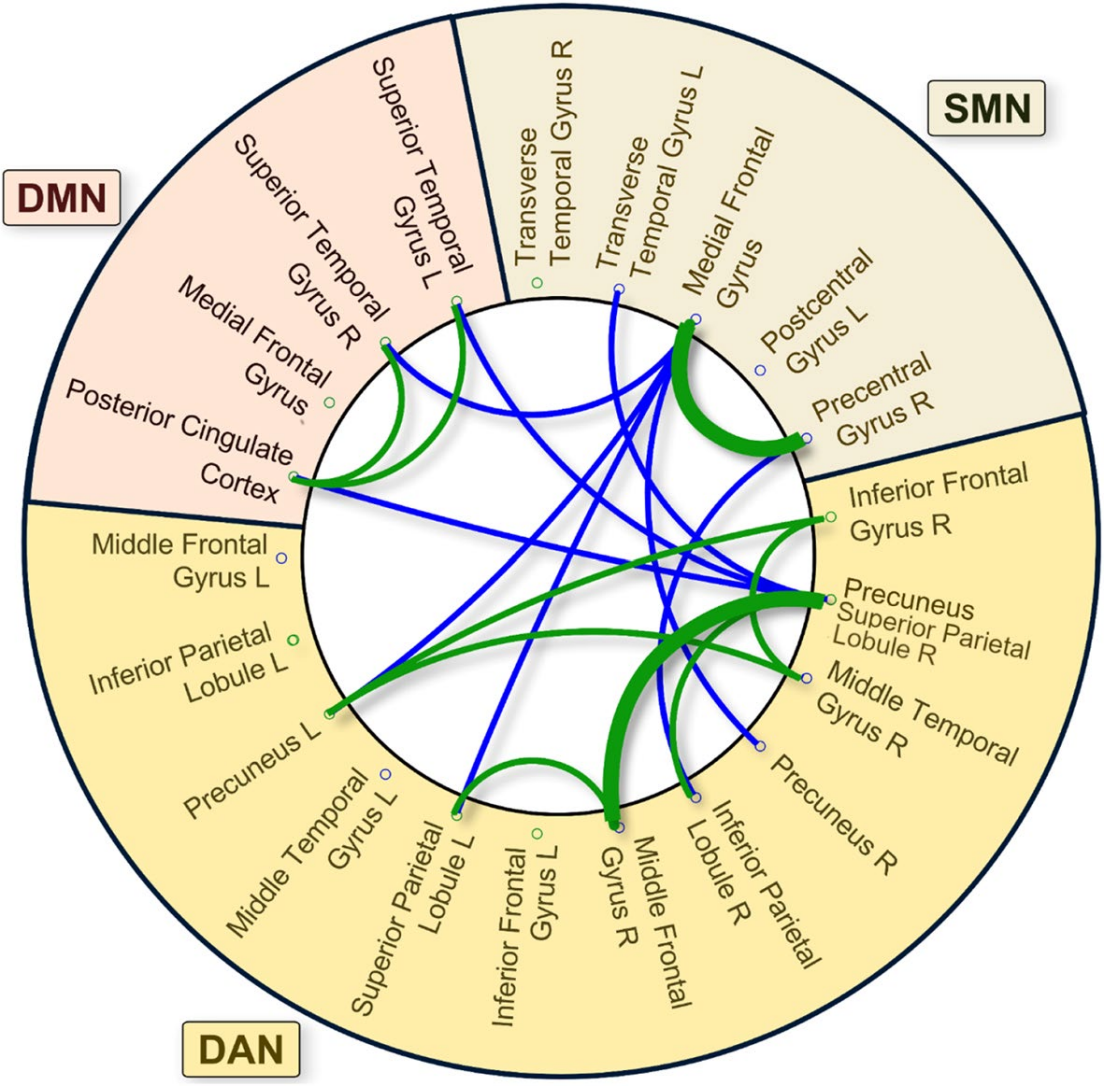
Summary of decreased connectivity within and between RSN in the ASD group relative to TD. Lines indicate significant decreased lagged phase synchronization for the ASD group relative to the TD group. Green lines represent decreased connectivity within RSNs, and blue lines represent decreased connectivity between RSNs. Thin line *p* < 0.05, thick line *p* < 0.01. L, left, R right

The ASD and TD groups also differed on connectivity between the 3-RSNs (Table 4, Fig. 3, and summary in Fig. 4). No difference was found for ASD > TD. For lagged phase synchronization between SMN and DAN, we identified significantly decreased connectivity in the ASD group relative to the TD group (1) between the medial frontal gyrus of the SMN and both the left superior parietal lobule (*t* = −3.667, *p* < 0.025) and the bilateral precuneus of the DAN (right precuneus: *t* = −3.611, *p* < 0.028; left precuneus: *t* = −3.459, *p* < 0.038), (2) between the left transverse temporal gyrus of the SMN and the right precuneus (superior parietal) of the DAN (*t* = −3.640, *p* < 0.026), and (3) between the right precentral gyrus of the SMN and the right inferior parietal lobule of the DAN (*t* = −3.561, *p* < 0.032). For SMN and DMN, we identified significantly decreased connectivity in the ASD group relative to the TD group between the medial frontal gyrus of the SMN and the right superior temporal gyrus of the DMN (*t* = −3.529, *p* < 0.015). For DAN and DMN connectivity, results revealed significantly decreased lagged phase synchronization in the ASD group relative to the TD group between the right precuneus (superior parietal) of the DAN and both the left superior temporal gyrus (*t* = −3.460, *p* < 0.033) and the posterior cingulate cortex of the DMN (*t* = −3.293, *p* < 0.05).

**Table 4:** Significant differences in between-RSN functional connectivity in the ASD group relative to the TD group

Regions	MNI coordinates			Regions	MNI coordinates			*t*	*p*
	*x*	*y*	*z*		*x*	*y*	*z*		
SMN and DAN									
*SMN*				*DAN*					
Medial frontal gyrus	−4	−33	66	Superior parietal lobule L	−8	−63	57	−3.667	<0.025
Transverse temporal gyrus L	−56	−23	12	Precuneus-superior parietal lobule R	8	−63	57	−3.640	<0.026
Medial frontal gyrus	−4	−33	66	Precuneus R	18	−69	51	−3.611	<0.028
Precentral gyrus R	34	−32	59	Inferior parietal lobule R	34	−38	44	−3.561	<0.032
Medial frontal gyrus	−4	−33	66	Precuneus L	−18	−69	51	−3.459	<0.038
SMN and DMN									
*SMN*				*DMN*					
Medial frontal gyrus	−4	−33	66	Superior temporal gyrus R	41	−60	29	−3.529	<0.015
DAN and DMN									
*DAN*				*DMN*					
Precuneus-superior parietal lobule R	8	−63	57	Superior temporal gyrus L	−41	−60	29	−3.460	<0.033
Precuneus-superior parietal lobule R	8	−63	57	Posterior cingulate cortex	0	−52	26	−3.293	<0.05

*SMN* sensorimotor network, *DAN* dorsal attention network, *DMN* default mode network, *L* left, *R* right

Backward stepwise regression analyses with age and ASD symptomatology (AQ) as individual predictors revealed no significant effect for the ASD group. However, we observed significant results in the TD group. Age was a significant predictor within the three 3-RSNs (SMN, DAN, and DMN) and between RSN functional connectivity (SMN-DAN and DAN-DMN). AQ was a significant predictor within 2-RSNs (DAN and DMN) and between RSN functional connectivity (DAN-SMN and DMN-SMN, see supplementary data for details).

## Discussion

The present study investigated sources and functional connectivity in the 3-RSN in the alpha frequency band, comparing participants with and without ASD, using lagged phase synchronization. We observed decreased connectivity within and between the DMN, the SMN, and the DAN in the ASD group compared to the TD group. We highlighted three principal hubs of dysconnectivity: the posterior cingulate cortex, the precuneus, and the medial frontal gyrus. We also showed reduced current source density in the alpha band mainly located in temporoparietal and somatosensory/medial areas considered as key nodes in the DMN, SMN, and DAN.

### Alpha band: a relevant marker for studying ASD atypicality

Our results showed decreased activity and connectivity in the alpha band in ASD participants. The alpha band is involved in controlling and regulating the information flow, both within and between brain networks, as it selectively inhibits task-irrelevant pathways [7, 70, 86]. Moreover, alpha activity seems to be associated with modulating sensory input [70, 87, 88], and alpha disturbances could lead to atypical sensory processing in ASD [89, 90]. Given together, this might suggest that individuals with ASD would have more difficulty integrating incoming information (bottom-up) at rest.

Increasing evidence from publications also shows that alpha activity plays a role in abnormal social and emotional stimuli processing in ASD [91, 92]. Given the alpha band involvement in attention, perception, and social cognition [6, 7, 31, 93], we speculated that alpha atypicality would participate in autistic symptoms. In this vein, Shephard et al. [94] recently showed that children with ASD who exhibit greater reductions in alpha power have more social communication difficulties.

In their review, Wang et al. [4] proposed that decreased alpha band may be related to an imbalance of excitatory (e.g., glutamatergic) and inhibitory (e.g., GABAergic) activity in ASD (also see [92, 95]). In particular, alpha desynchronization may be linked to increased neural excitability/decreased neural inhibition [7]. Previous studies in ASD reported abnormal cortical inhibitory interneurons [96, 97] and altered glutamatergic levels [98, 99], associated with atypical sensory processing in ASD [90, 100]. The excitation/inhibition imbalance increases neural noise in ASD and may lead to atypical sensory processing and under-responsiveness to behaviorally relevant stimuli in participants with ASD [89].

Thus, our data point out that excitation/inhibition imbalance highlighted by a decrease in alpha activity increases neural noise in ASD. This may result in integration difficulties affecting both external information (sensory processing, bottom-up) and internal information (mental representations, top-down) with an effect on the representation of oneself in the world and the shifting of mental representations [101–103].

### Functional connectivity within resting-state networks

We found reduced lagged phase synchronization within the SMN, DAN, and DMN in the ASD group compared to the TD group, possibly reflecting an imbalance between bottom-up and top-down information integration explained as follows. This reduction is correlated with neither age nor symptom severity in the ASD group. However, we observed mainly an age effect on connectivity within the DAN, SMN, and DMN in the TD group supporting a developmental effect on RSNs. The same pattern is observed for between RSNs. We can hypothesis particularities in connectivity observed in autism are stable over time.

First, we highlighted underconnectivity within the SMN, between the medial frontal gyrus and the right precentral gyrus. A recent fMRI study also showed reduced functional connectivity among visual association, somatosensory, and motor networks [104]. This decreased functional connectivity between somatosensory and motor networks seems to be consistent with the atypical multisensory and motor integration observed in individuals with ASD and could participate in the core symptoms of ASD [105, 106]. Dysconnectivity within the SMN may result in an atypical integration of the external sensory environment and might be interpreted as an index of increased sensitivity to sensory information, which is in line with the enhanced perceptual functioning theory [107]. Therefore, aberrant functional connectivity in the SMN may explain multisensory and motor integration deficits, which play a crucial role in developing imitation, motor communication, and social skills [108, 109], all impaired in ASD.

Second, in line with previously findings reported in fMRI studies [41, 110–112] and MEG studies for the gamma band [38], participants with ASD exhibited decreased connectivity within the DMN, particularly involving the posterior cingulate cortex and right and left superior temporal gyrus. Li et al. showed that underconnectivity involving the posterior cingulate cortex, insula, and superior temporal gyrus was negatively associated with ASD symptom severity measured by ADOS [113]. These DMN perturbations may profoundly modify internal information, including memory, mental scene construction, or the sense of self, and act as a significant contributor to social dysfunction (see [41] for review).

Finally, in agreement with previously reported results in fMRI by Bi et al. [50] and Farrant and Uddin [51], we found dysconnectivity within several DAN regions for participants with ASD relative to the TD group. We observed the main underconnectivity between the right precuneus-superior parietal lobule and right middle frontal gyrus, consistent with difficulties in attention regulation and shifting between external and internal stimuli that may directly impact self-information integration. More broadly, as the DAN is involved in regulating exogenous goal-directed top-down processing [49], it would be possible that this atypical connectivity might participate in shifting difficulties associated with autistic symptoms [114].

### Functional connectivity between resting-state networks

We showed decrease between-network connectivity between SMN, DAN, and DMN in the ASD group relative to the TD group. Importantly, the communication among brain regions belonging to the SMN, DAN, and DMN could be related to the ability to switch between external and internal information sources.

The underconnectivity observed in the ASD group between the SMN and DAN may generate delays in perceptual input and information transmission, thereby compromising the integration of information from the environment [40, 115]. In ASD, the attentional focus seems to be less oriented to the external information, especially when directly related to own’s body representation. Therefore, these results shed light on the atypical perception that may focus on details in ASD [107, 116].

Moreover, the underconnectivity we observed between the right superior temporal gyrus in the DMN and the medial frontal gyrus in the SMN could participate in the atypical integration of external sensory information (SMN) and bind this external information to internal information (DMN), which may modify the perception of environmental stimuli related to the self [117]. Hence, an atypical representation of the body and its location in space provided by the SMN may participate in an atypical sense of self [118, 119].

Finally, the underconnectivity we observed between DAN (precuneus-superior parietal lobule) and DMN (superior temporal gyrus and posterior cingulate cortex) provides additional arguments pointing to a communication deficit in ASD. This decreased connectivity suggests difficulties switching between externally oriented attention (the world) and internally oriented thoughts (representations of the world and the self). Furthermore, studies have shown that decreased connectivity between the precuneus and temporal gyrus is correlated with the ADOS social score [101], highlighting the importance of these regions in ASD symptomatology.

The reduced connectivity between these 3-RSNs seems to imply an atypical integration of both information from the external environment via the sensory organs (bottom-up) and top-down mental representations of the environment (memories, beliefs or expectations, top-down) but also to switch between these external and internal information [5]. The difficulty in integrating incoming sensory information to previously stored information could be linked to the weak central coherence theory [120]. The sensory over-responsivity may lead to a chronic state of hyperarousal at rest [121] that would impact the internal organization.

The present study also highlighted three key nodes, which presented decreased current source density. First, the medial frontal gyrus was associated with reduced connectivity within and between the 3-RSNs, suggesting atypical multisensory integration, feeling of body ownership [122], and imitation skills, which play a role in social skills [108, 109]. Second, the precuneus is involved in spatial functions of self and the spatial environment and may contribute to the altered sense of self and agency in ASD [118, 119]. The precuneus, with a more lateral cortex, is also involved in attentional shifting from low- (e.g., simple stimulus) to high-level processing (shifting mental representations; e.g., theory of mind [123, 124]). The precuneus associated with the posterior cingulate cortex contributes to self/other referential processing [125, 126] and the monitoring of the environment [127]. Third, the posterior cingulate cortex’s underconnectivity with the superior temporal gyrus and precuneus may account for difficulty regulating and balancing the focus of attention on internal or external thoughts [128], arousal and awareness [129], and self-referential thought [130] and affect the stability of the brain network over time (*whole-brain metastability* [131];). Given their involvement, abnormalities in these brain regions may significantly impact social disabilities in autism.

Beyond, from a broader perspective, our results might be better understood though the lens of the embodied cognition framework. Embodied cognition refers to the theory that the environment and the body (e.g., sensorimotor information) influence cognition (e.g., memory, mental simulation, representation, and attention) with a *bottom-up* process and, reversely, how cognition influences the body through a *top-down* process. Specifically, the SMN and its relationship to the DMN and DAN may be an important brain correlate of the perception-action loop [132]. In the case of ASD individuals, an impairment in the communication between and within the SMN may be responsible to poor integration of sensorimotor information [133–135]. Reduced functional connectivity may modify the conscious perception of the external world in relation to self-referential processes, with possible consequences on social skills [40, 136–138].

### Limitations

We used sLORETA to measure cerebral activity and identify brain regions, but this technique has spatial limitations. As such, other methods would be needed to precise the spatial characteristics of our results. Second, we have chosen a wide period of adolescence, from 10 to 25 years, and it would be relevant to examine specific neurodevelopmental changes between early and late adolescence. Such a study would enable the results to be replicated with a larger sample size. Hypotheses have been formulated on the cognitive difficulties observed in autism but it would be very interesting to confirm them by directly analyzing the relation between connectivity deficits and ASD symptoms, especially social difficulties. High heterogeneity was described in ASD and other studies would be needed to precise for example the relation between hypo- or hyper-sensitivity to sensory stimuli and connectivity. Finally, our population was composed of high-functioning participants, limiting generalization to adolescents across the spectrum.

## Conclusion

We report reduced functional connectivity within and between DMN, SMN, and DAN in the ASD group relative to the TD group in the alpha band. Impaired neural communication within and between the 3-RSN may lead to altered integration and switching between externally oriented information and internally oriented thoughts. Indeed, hypoconnectivity analyses revealed atypicality in integrative brain regions that may account for difficulties in (1) perception (external): somatosensory integration of the environment, (2) cognition (memory, mental representation, attention, etc.) integration of internal representations, and (3) reciprocal influence between external environmental stimuli to internal representations (bottom-up process) and, reversely, between cognition and the body through a *top-down* process. These possible impairments in the embodiment are interesting to consider and might participate in social abilities: high-level integration required for related self-processing, notably interaction (e.g., nonverbal communication such as gestures, facial expressions, and modulation of timing and intonation of speech) and theory of mind, which requires individuals to switch from a self-perspective to another perspective.

## Supplementary Information

**Additional file 1. ** Significant differences in current source density in alpha band for ASD and TD groups (*p* < 0.05). For each region that showed significant decrease in current source density, the number of voxels, the localization of the maximum statistical peak difference, and the involvement in a RSN of interest for this region were indicated. The names of the regions are the labels given by sLORETA for the corresponding coordinates.

**Additional file 2.** Backward stepwise regression analyses with age and ASD symptomatology (AQ) as individual predictors on connectivity values (within and between-RSN functional connectivity) that were significantly different between groups. Significant results were obtained only for the TD group. SMN: sensorimotor network; DAN: dorsal attention network; DMN: default mode network; L: left, R: right. RSNs: Resting State Networks.

## Data Availability

The datasets used and/or analyzed in the current study are available from the corresponding author upon reasonable request.

## Abbreviations

ADOS: Autism Diagnostic Observation Schedule
AQ: Autism spectrum quotient
ASD: Autism spectrum disorder
DAN: Dorsal attention network
DMN: Default mode network
DSM: Diagnostic and Statistical Manual of Mental Disorders
EEG: Electroencephalography
fMRI: Functional magnetic resonance imaging
MEG: Magnetoencephalography
MNI: Montreal Neurological Institute
ROIs: Regions of interest
RS: Resting state
RSN: Resting-state network
sLORETA: Standardized low-resolution brain electromagnetic tomography
SMN: Sensorimotor network
SnPM: Statistical non-parametric mapping
TD: Typically developing

## References

[1] American Psychiatric Association. Diagnostic and statistical manual of mental disorders [Internet]. Fifth edition. Arlington, VA: American Psychiatric Association; 2013. Available from: http://psychiatryonline.org/doi/book/10.1176/appi.books.9780890425596. Cited 2016 Nov 2.

[2] Hull L, Petrides KV, Allison C, Smith P, Baron-Cohen S, Lai MC (2017). “Putting on My Best Normal”: social camouflaging in adults with autism spectrum conditions. J Autism Dev Disord.

[3] O’Reilly C, Lewis JD, Elsabbagh M (2017). Is functional brain connectivity atypical in autism? A systematic review of EEG and MEG studies. PLoS One.

[4] Wang J, Barstein J, Ethridge LE, Mosconi MW, Takarae Y, Sweeney JA (2013). Resting state EEG abnormalities in autism spectrum disorders. J Neurodev Disord.

[5] Christoff K, Irving ZC, Fox KCR, Spreng RN, Andrews-Hanna JR (2016). Mind-wandering as spontaneous thought: a dynamic framework. Nat Rev Neurosci.

[6] Edgar JC, Heiken K, Chen YH, Herrington JD, Chow V, Liu S (2015). Resting-state alpha in autism spectrum disorder and alpha associations with thalamic volume. J Autism Dev Disord.

[7] Klimesch W, Sauseng P, Hanslmayr S (2007). EEG alpha oscillations: the inhibition-timing hypothesis. Brain Res Rev.

[8] Neuper C, Pfurtscheller G (2001). Event-related dynamics of cortical rhythms: frequency-specific features and functional correlates. Int J Psychophysiol Off J Int Organ Psychophysiol.

[9] Pfurtscheller G, Stancák A, Neuper C (1996). Event-related synchronization (ERS) in the alpha band--an electrophysiological correlate of cortical idling: a review. Int J Psychophysiol Off J Int Organ Psychophysiol.

[10] Jokisch D, Jensen O (2007). Modulation of gamma and alpha activity during a working memory task engaging the dorsal or ventral stream. J Neurosci.

[11] Boiten F, Sergeant J, Geuze R (1992). Event-related desynchronization: the effects of energetic and computational demands. Electroencephalogr Clin Neurophysiol.

[12] Klimesch W (1999). EEG alpha and theta oscillations reflect cognitive and memory performance: a review and analysis. Brain Res Rev.

[13] Boersma M, Kemner C, de Reus MA, Collin G, Snijders TM, Hofman D (2013). Disrupted functional brain networks in autistic toddlers. Brain Connect.

[14] Carson AM, Salowitz NMG, Scheidt RA, Dolan BK, Van Hecke AV (2014). Electroencephalogram coherence in children with and without autism spectrum disorders: decreased interhemispheric connectivity in autism. Autism Res Off J Int Soc Autism Res.

[15] Clarke AR, Barry RJ, Indraratna A, Dupuy FE, McCarthy R, Selikowitz M (2016). EEG activity in children with Asperger’s syndrome. Clin Neurophysiol Off J Int Fed Clin Neurophysiol.

[16] Coben R, Clarke AR, Hudspeth W, Barry RJ (2008). EEG power and coherence in autistic spectrum disorder. Clin Neurophysiol.

[17] Duffy FH, Als H (2012). A stable pattern of EEG spectral coherence distinguishes children with autism from neuro-typical controls - a large case control study. BMC Med.

[18] Elhabashy H, Raafat O, Afifi L, Raafat H, Abdullah K (2015). Quantitative EEG in autistic children. Egypt J Neurol Psychiatry Neurosurg.

[19] Jaime M, McMahon CM, Davidson BC, Newell LC, Mundy PC, Henderson HA (2016). Brief report: Reduced temporal-central EEG alpha coherence during joint attention perception in adolescents with autism spectrum disorder. J Autism Dev Disord.

[20] Lushchekina EA, Podreznaya ED, Lushchekin VS, Strelets VB (2012). A comparative EEG study in normal and autistic children. Neurosci Behav Physiol.

[21] Matlis S, Boric K, Chu CJ, Kramer MA (2015). Robust disruptions in electroencephalogram cortical oscillations and large-scale functional networks in autism. BMC Neurol.

[22] Murias M, Webb SJ, Greenson J, Dawson G (2007). Resting state cortical connectivity reflected in EEG coherence in individuals with autism. Biol Psychiatry.

[23] Cantor DS, Thatcher RW, Hrybyk M, Kaye H (1986). Computerized EEG analyses of autistic children. J Autism Dev Disord.

[24] Orekhova EV, Elsabbagh M, Jones EJ, Dawson G, Charman T, Johnson MH (2014). EEG hyper-connectivity in high-risk infants is associated with later autism. J Neurodev Disord.

[25] Sheikhani A, Behnam H, Mohammadi MR, Noroozian M, Mohammadi M (2012). Detection of abnormalities for diagnosing of children with autism disorders using of quantitative electroencephalography analysis. J Med Syst.

[26] Maxwell CR, Villalobos ME, Schultz RT, Herpertz-Dahlmann B, Konrad K, Kohls G (2015). Atypical laterality of resting gamma oscillations in autism spectrum disorders. J Autism Dev Disord.

[27] Daoust AM, Limoges E, Bolduc C, Mottron L, Godbout R (2004). EEG spectral analysis of wakefulness and REM sleep in high functioning autistic spectrum disorders. Clin Neurophysiol Off J Int Fed Clin Neurophysiol.

[28] Dumas G, Soussignan R, Hugueville L, Martinerie J, Nadel J (2014). Revisiting mu suppression in autism spectrum disorder. Brain Res.

[29] Barttfeld P, Wicker B, Cukier S, Navarta S, Lew S, Sigman M (2011). A big-world network in ASD: dynamical connectivity analysis reflects a deficit in long-range connections and an excess of short-range connections. Neuropsychologia..

[30] Han YMY, Chan AS (2016). Disordered cortical connectivity underlies the executive function deficits in children with autism spectrum disorders. Res Dev Disabil.

[31] Mathewson KJ, Jetha MK, Drmic IE, Bryson SE, Goldberg JO, Schmidt LA (2012). Regional EEG alpha power, coherence, and behavioral symptomatology in autism spectrum disorder. Clin Neurophysiol.

[32] Khan S, Gramfort A, Shetty NR, Kitzbichler MG, Ganesan S, Moran JM (2013). Local and long-range functional connectivity is reduced in concert in autism spectrum disorders. Proc Natl Acad Sci U S A.

[33] Wass S (2011). Distortions and disconnections: disrupted brain connectivity in autism. Brain Cogn.

[34] Gandal MJ, Edgar JC, Ehrlichman RS, Mehta M, Roberts TPL, Siegel SJ (2010). Validating γ oscillations and delayed auditory responses as translational biomarkers of autism. Biol Psychiatry.

[35] Rojas DC, Maharajh K, Teale P, Rogers SJ (2008). Reduced neural synchronization of gamma-band MEG oscillations in first-degree relatives of children with autism. BMC Psychiatry.

[36] Nunez PL, Silberstein RB, Cadusch PJ, Wijesinghe RS, Westdorp AF, Srinivasan R (1994). A theoretical and experimental study of high resolution EEG based on surface Laplacians and cortical imaging. Electroencephalogr Clin Neurophysiol.

[37] Coben R, Mohammad-Rezazadeh I, Cannon RL. Using quantitative and analytic EEG methods in the understanding of connectivity in autism spectrum disorders: a theory of mixed over- and under-connectivity. Front Hum Neurosci. 2014;8 Available from: http://www.ncbi.nlm.nih.gov/pmc/articles/PMC3935255/. Cited 2015 Apr 21. 10.3389/fnhum.2014.00045 PMC3935255 24616679

[38] Lajiness-O’Neill R, Brennan JR, Moran JE, Richard AE, Flores AM, Swick C (2018). Patterns of altered neural synchrony in the default mode network in autism spectrum disorder revealed with magnetoencephalography (MEG): relationship to clinical symptomatology. Autism Res Off J Int Soc Autism Res.

[39] Gusnard DA, Akbudak E, Shulman GL, Raichle ME (2001). Medial prefrontal cortex and self-referential mental activity: relation to a default mode of brain function. Proc Natl Acad Sci U S A.

[40] Weng SJ, Wiggins JL, Peltier SJ, Carrasco M, Risi S, Lord C (2010). Alterations of resting state functional connectivity in the default network in adolescents with autism spectrum disorders. Brain Res.

[41] Padmanabhan A, Lynch CJ, Schaer M, Menon V (2017). The default mode network in autism. Biol Psychiatry Cogn Neurosci Neuroimaging.

[42] Perry W, Minassian A, Lopez B, Maron L, Lincoln A (2007). Sensorimotor gating deficits in adults with autism. Biol Psychiatry.

[43] Takarae Y, Minshew NJ, Luna B, Sweeney JA (2007). Atypical involvement of frontostriatal systems during sensorimotor control in autism. Psychiatry Res Neuroimaging.

[44] Travers BG, Kana RK, Klinger LG, Klein CL, Klinger MR (2015). Motor learning in individuals with autism spectrum disorder: activation in superior parietal lobule related to learning and repetitive behaviors. Autism Res Off J Int Soc Autism Res.

[45] Boyd BA, Baranek GT, Sideris J, Poe MD, Watson LR, Patten E (2010). Sensory features and repetitive behaviors in children with autism and developmental delays. Autism Res Off J Int Soc Autism Res.

[46] Cascio CJ, Woynaroski T, Baranek GT, Wallace MT (2016). Toward an interdisciplinary approach to understanding sensory function in autism spectrum disorder. Autism Res.

[47] Foss-Feig JH, Heacock JL, Cascio CJ (2012). Tactile responsiveness patterns and their association with core features in autism spectrum disorders. Res Autism Spectr Disord.

[48] Lim YH, Partridge K, Girdler S, Morris SL (2017). Standing postural control in individuals with autism spectrum disorder: systematic review and meta-analysis. J Autism Dev Disord.

[49] Corbetta M, Shulman GL (2002). Control of goal-directed and stimulus-driven attention in the brain. Nat Rev Neurosci.

[50] Xan B, Zhao J, Xu Q, Sun Q, Wang Z. Abnormal functional connectivity of resting state network detection based on linear ICA analysis in autism spectrum disorder. Front Physiol. 2018;9 Available from: https://www.frontiersin.org/articles/10.3389/fphys.2018.00475/full. Cited 2019 Jul 31. 10.3389/fphys.2018.00475 PMC5952255 29867534

[51] Farrant K, Uddin LQ (2016). Atypical developmental of dorsal and ventral attention networks in autism. Dev Sci.

[52] Palva S, Palva JM (2011). Functional roles of alpha-band phase synchronization in local and large-scale cortical networks. Front Psychol.

[53] Sadaghiani S, Scheeringa R, Lehongre K, Morillon B, Giraud AL, Kleinschmidt A (2010). Intrinsic connectivity networks, alpha oscillations, and tonic alertness: a simultaneous electroencephalography/functional magnetic resonance imaging study. J Neurosci.

[54] van Ede F, de Lange F, Jensen O, Maris E (2011). Orienting attention to an upcoming tactile event involves a spatially and temporally specific modulation of sensorimotor alpha- and beta-band oscillations. J Neurosci.

[55] Haegens S, Nácher V, Luna R, Romo R, Jensen O (2011). α-Oscillations in the monkey sensorimotor network influence discrimination performance by rhythmical inhibition of neuronal spiking. Proc Natl Acad Sci.

[56] Jones SR, Kerr CE, Wan Q, Pritchett DL, Hämäläinen M, Moore CI (2010). Cued spatial attention drives functionally relevant modulation of the mu rhythm in primary somatosensory cortex. J Neurosci.

[57] Canuet L, Ishii R, Pascual-Marqui RD, Iwase M, Kurimoto R, Aoki Y (2011). Resting-state EEG source localization and functional connectivity in schizophrenia-like psychosis of epilepsy. PLoS One.

[58] Logothetis NK (2008). What we can do and what we cannot do with fMRI. Nature..

[59] Cerliani L, Mennes M, Thomas RM, Martino AD, Thioux M, Keysers C (2015). Increased functional connectivity between subcortical and cortical resting-state networks in autism spectrum disorder. JAMA Psychiatry.

[60] Nomi JS, Uddin LQ (2015). Developmental changes in large-scale network connectivity in autism. NeuroImage Clin.

[61] Barber AD, Caffo BS, Pekar JJ, Mostofsky SH (2013). Developmental changes in within- and between-network connectivity between late childhood and adulthood. Neuropsychologia..

[62] Bassett DS, Yang M, Wymbs NF, Grafton ST (2015). Learning-induced autonomy of sensorimotor systems. Nat Neurosci.

[63] Sawyer SM, Azzopardi PS, Wickremarathne D, Patton GC (2018). The age of adolescence. Lancet Child Adolesc Health.

[64] Lord C, Rutter M, Le Couteur A (1994). Autism diagnostic interview-revised: a revised version of a diagnostic interview for caregivers of individuals with possible pervasive developmental disorders. J Autism Dev Disord.

[65] Lord C, Risi S, Lambrecht L, Cook EH, Leventhal BL, DiLavore PC (2000). The autism diagnostic observation schedule-generic: a standard measure of social and communication deficits associated with the spectrum of autism. J Autism Dev Disord.

[66] Baron-Cohen S, Wheelwright S, Skinner R, Martin J, Clubley E (2001). The autism-spectrum quotient (AQ): evidence from Asperger syndrome/high-functioning autism, males and females, scientists and mathematicians. J Autism Dev Disord.

[67] Wechsler D (2003). Wechsler intelligence scale for children – fourth edition (WISC-IV).

[68] Wechsler D (2008). Wechsler Adult Intelligence Scale-fourth edition.

[69] Tucker DM (1993). Spatial sampling of head electrical fields: the geodesic sensor net. Electroencephalogr Clin Neurophysiol.

[70] Jensen O, Mazaheri A. Shaping functional architecture by oscillatory alpha activity: gating by inhibition. Front Hum Neurosci. 2010;4 Available from: https://www.ncbi.nlm.nih.gov/pmc/articles/PMC2990626/. Cited 2019 Sep 21. 10.3389/fnhum.2010.00186 PMC2990626 21119777

[71] Palva S, Palva JM (2007). New vistas for alpha-frequency band oscillations. Trends Neurosci.

[72] Damoiseaux JS, Rombouts SARB, Barkhof F, Scheltens P, Stam CJ, Smith SM (2006). Consistent resting-state networks across healthy subjects. Proc Natl Acad Sci U S A.

[73] Miskovic V, Ma X, Chou CA, Fan M, Owens M, Sayama H (2015). Developmental changes in spontaneous electrocortical activity and network organization from early to late childhood. NeuroImage..

[74] Ferree TC, Luu P, Russell GS, Tucker DM (2001). Scalp electrode impedance, infection risk, and EEG data quality. Clin Neurophysiol Off J Int Fed Clin Neurophysiol.

[75] Pascual-Marqui RD, Lehmann D, Koukkou M, Kochi K, Anderer P, Saletu B (2011). Assessing interactions in the brain with exact low-resolution electromagnetic tomography. Philos Transact A Math Phys Eng Sci.

[76] Billeci L, Sicca F, Maharatna K, Apicella F, Narzisi A, Campatelli G, et al. On the application of quantitative EEG for characterizing autistic brain: a systematic review. Front Hum Neurosci. 2013;7 Available from: http://journal.frontiersin.org/article/10.3389/fnhum.2013.00442/full. Cited 2017 May 26. 10.3389/fnhum.2013.00442 PMC3733024 23935579

[77] Freeman W, Quiroga RQ (2012). Imaging brain function with EEG: advanced temporal and spatial analysis of electroencephalographic signals.

[78] Mathewson KJ, Hashemi A, Sheng B, Sekuler AB, Bennett PJ, Schmidt LA (2015). Regional electroencephalogram (EEG) alpha power and asymmetry in older adults: a study of short-term test–retest reliability. Front Aging Neurosci.

[79] Pascual-Marqui RD, Esslen M, Kochi K, Lehmann D (2002). Functional imaging with low-resolution brain electromagnetic tomography (LORETA): a review. Methods Find Exp Clin Pharmacol.

[80] Pascual-Marqui RD, Michel CM, Lehmann D (1994). Low resolution electromagnetic tomography: a new method for localizing electrical activity in the brain. Int J Psychophysiol Off J Int Organ Psychophysiol.

[81] Gianotti LRR, Dahinden FM, Baumgartner T, Knoch D (2019). Understanding individual differences in domain-general prosociality: a resting EEG study. Brain Topogr.

[82] Holmes AP, Blair RC, Watson JD, Ford I (1996). Nonparametric analysis of statistic images from functional mapping experiments. J Cereb Blood Flow Metab Off J Int Soc Cereb Blood Flow Metab.

[83] Nichols TE, Holmes AP (2002). Nonparametric permutation tests for functional neuroimaging: a primer with examples. Hum Brain Mapp.

[84] Wu CW, Gu H, Lu H, Stein EA, Chen JH, Yang Y (2008). Frequency specificity of functional connectivity in brain networks. NeuroImage..

[85] Yeo BTT, Krienen FM, Sepulcre J, Sabuncu MR, Lashkari D, Hollinshead M (2011). The organization of the human cerebral cortex estimated by intrinsic functional connectivity. J Neurophysiol.

[86] Zumer JM, Scheeringa R, Schoffelen JM, Norris DG, Jensen O (2014). Occipital alpha activity during stimulus processing gates the information flow to object-selective cortex. PLoS Biol.

[87] Klimesch W (2012). Alpha-band oscillations, attention, and controlled access to stored information. Trends Cogn Sci.

[88] Mathewson KE, Lleras A, Beck DM, Fabiani M, Ro T, Gratton G (2011). Pulsed out of awareness: EEG alpha oscillations represent a pulsed-inhibition of ongoing cortical processing. Front Psychol.

[89] Keehn B, Westerfield M, Müller RA, Townsend J (2017). Autism, attention, and alpha oscillations: an electrophysiological study of attentional capture. Biol Psychiatry Cogn Neurosci Neuroimaging.

[90] Pierce S, Kadlaskar G, Edmondson DA, McNally Keehn R, Dydak U, Keehn B (2021). Associations between sensory processing and electrophysiological and neurochemical measures in children with ASD: an EEG-MRS study. J Neurodev Disord.

[91] Rippon G, Brock J, Brown C, Boucher J (2007). Disordered connectivity in the autistic brain: challenges for the “new psychophysiology”. Int J Psychophysiol Off J Int Organ Psychophysiol.

[92] Rubenstein JLR, Merzenich MM (2003). Model of autism: increased ratio of excitation/inhibition in key neural systems. Genes Brain Behav.

[93] Dickinson A, DiStefano C, Senturk D, Jeste SS (2018). Peak alpha frequency is a neural marker of cognitive function across the autism spectrum. Eur J Neurosci.

[94] Shephard E, Tye C, Ashwood KL, Azadi B, Asherson P, Bolton PF (2018). Resting-state neurophysiological activity patterns in young people with ASD, ADHD, and ASD + ADHD. J Autism Dev Disord.

[95] Sohal VS, Rubenstein JLR (2019). Excitation-inhibition balance as a framework for investigating mechanisms in neuropsychiatric disorders. Mol Psychiatry.

[96] Casanova MF, Buxhoeveden DP, Switala AE, Roy E (2002). Minicolumnar pathology in autism. Neurology..

[97] Levitt P, Eagleson KL, Powell EM (2004). Regulation of neocortical interneuron development and the implications for neurodevelopmental disorders. Trends Neurosci.

[98] Tebartz van Elst L, Maier S, Fangmeier T, Endres D, Mueller GT, Nickel K (2014). Disturbed cingulate glutamate metabolism in adults with high-functioning autism spectrum disorder: evidence in support of the excitatory/inhibitory imbalance hypothesis. Mol Psychiatry.

[99] Zheng Z, Zhu T, Qu Y, Mu D (2016). Blood glutamate levels in autism spectrum disorder: a systematic review and meta-analysis. PLoS One.

[100] He JL, Oeltzschner G, Mikkelsen M, Deronda A, Harris AD, Crocetti D (2021). Region-specific elevations of glutamate + glutamine correlate with the sensory symptoms of autism spectrum disorders. Transl Psychiatry.

[101] Cheng W, Rolls ET, Gu H, Zhang J, Feng J (2015). Autism: reduced connectivity between cortical areas involved in face expression, theory of mind, and the sense of self. Brain J Neurol.

[102] Lau WKW, Leung MK, Lau BWM (2019). Resting-state abnormalities in autism spectrum disorders: a meta-analysis. Sci Rep.

[103] Lombardo MV, Chakrabarti B, Bullmore ET, Sadek SA, Pasco G, Wheelwright SJ (2010). Atypical neural self-representation in autism. Brain J Neurol.

[104] Oldehinkel M, Mennes M, Marquand A, Charman T, Tillmann J, Ecker C (2019). Altered connectivity between cerebellum, visual, and sensory-motor networks in autism spectrum disorder: results from the EU-AIMS Longitudinal European Autism Project. Biol Psychiatry Cogn Neurosci Neuroimaging.

[105] Ben-Sasson A, Hen L, Fluss R, Cermak SA, Engel-Yeger B, Gal E (2009). A meta-analysis of sensory modulation symptoms in individuals with autism spectrum disorders. J Autism Dev Disord.

[106] Marco EJ, Hinkley LBN, Hill SS, Nagarajan SS (2011). Sensory processing in autism: a review of neurophysiologic findings. Pediatr Res.

[107] Mottron L, Dawson M, Soulières I, Hubert B, Burack J (2006). Enhanced perceptual functioning in autism: an update, and eight principles of autistic perception. J Autism Dev Disord.

[108] Edwards LA (2014). A meta-analysis of imitation abilities in individuals with autism spectrum disorders. Autism Res.

[109] Williams JHG, Whiten A, Singh T (2004). A systematic review of action imitation in autistic spectrum disorder. J Autism Dev Disord.

[110] Kennedy DP, Courchesne E (2008). The intrinsic functional organization of the brain is altered in autism. NeuroImage..

[111] Uddin LQ (2011). The self in autism: an emerging view from neuroimaging. Neurocase..

[112] Washington SD, Gordon EM, Brar J, Warburton S, Sawyer AT, Wolfe A (2014). Dysmaturation of the default mode network in autism. Hum Brain Mapp.

[113] Li Q, Becker B, Jiang X, Zhao Z, Zhang Q, Yao S (2019). Decreased interhemispheric functional connectivity rather than corpus callosum volume as a potential biomarker for autism spectrum disorder. Cortex..

[114] Keehn B, Müller RA, Townsend J (2013). Atypical attentional networks and the emergence of autism. Neurosci Biobehav Rev.

[115] Belmonte MK, Allen G, Beckel-Mitchener A, Boulanger LM, Carper RA, Webb SJ (2004). Autism and abnormal development of brain connectivity. J Neurosci.

[116] Martínez K, Martínez-García M, Marcos-Vidal L, Janssen J, Castellanos FX, Pretus C (2020). Sensory-to-cognitive systems integration is associated with clinical severity in autism spectrum disorder. J Am Acad Child Adolesc Psychiatry.

[117] Boly M, Balteau E, Schnakers C, Degueldre C, Moonen G, Luxen A (2007). Baseline brain activity fluctuations predict somatosensory perception in humans. Proc Natl Acad Sci U S A.

[118] Cavanna AE, Trimble MR (2006). The precuneus: a review of its functional anatomy and behavioural correlates. Brain..

[119] Lyons V, Fitzgerald M. Atypical sense of self in autism spectrum disorders: a neuro-cognitive perspective. Recent Adv Autism Spectr Disord - Vol I. 2013; Available from: https://www.intechopen.com/books/recent-advances-in-autism-spectrum-disorders-volume-i/atypical-sense-of-self-in-autism-spectrum-disorders-a-neuro-cognitive-perspective. Cited 2019 Aug 13.

[120] Happé F, Frith U (2006). The weak coherence account: detail-focused cognitive style in autism spectrum disorders. J Autism Dev Disord.

[121] Arora I, Bellato A, Ropar D, Hollis C, Groom MJ (2021). Is autonomic function during resting-state atypical in Autism: a systematic review of evidence. Neurosci Biobehav Rev.

[122] Ehrsson HH, Holmes NP, Passingham RE (2005). Touching a rubber hand: feeling of body ownership is associated with activity in multisensory brain areas. J Neurosci.

[123] Le TH, Pardo JV, Hu X (1998). 4 T-fMRI study of nonspatial shifting of selective attention: cerebellar and parietal contributions. J Neurophysiol.

[124] Nagahama Y, Okada T, Katsumi Y, Hayashi T, Yamauchi H, Sawamoto N (1999). Transient neural activity in the medial superior frontal gyrus and precuneus time locked with attention shift between object features. NeuroImage..

[125] Burrows CA, Laird AR, Uddin LQ (2016). Functional connectivity of brain regions for self- and other-evaluation in children, adolescents and adults with autism. Dev Sci.

[126] Zhao W, Luo L, Li Q, Kendrick KM. What can psychiatric disorders tell us about neural processing of the self? Front Hum Neurosci. 2013;7 Available from: https://www.ncbi.nlm.nih.gov/pmc/articles/PMC3744079/. Cited 2019 Aug 5. 10.3389/fnhum.2013.00485 PMC3744079 23966936

[127] Qin P, Northoff G (2011). How is our self related to midline regions and the default-mode network?. NeuroImage..

[128] Leech R, Kamourieh S, Beckmann CF, Sharp DJ (2011). Fractionating the default mode network: distinct contributions of the ventral and dorsal posterior cingulate cortex to cognitive control. J Neurosci.

[129] Vogt BA, Laureys S (2005). Posterior cingulate, precuneal and retrosplenial cortices: cytology and components of the neural network correlates of consciousness. Prog Brain Res.

[130] Brewer JA, Garrison KA, Whitfield-Gabrieli S. What about the “Self” is processed in the posterior cingulate cortex? Front Hum Neurosci. 2013;7 Available from: https://www.ncbi.nlm.nih.gov/pmc/articles/PMC3788347/. Cited 2019 Sep 21. 10.3389/fnhum.2013.00647 PMC3788347 24106472

[131] Leech R, Sharp DJ (2014). The role of the posterior cingulate cortex in cognition and disease. Brain J Neurol.

[132] Gibson JJ (2014). The ecological approach to visual perception: classic edition.

[133] De Jaegher H (2013). Embodiment and sense-making in autism. Front Integr Neurosci.

[134] Eigsti IM (2013). A review of embodiment in autism spectrum disorders. Front Psychol.

[135] Frith U, Hill EL, Klin A, Jones W, Schultz R, Volkmar F (2003). The enactive mind, or from actions to cognition: lessons from autism. Philos Trans R Soc Lond Ser B Biol Sci.

[136] Assaf M, Jagannathan K, Calhoun VD, Miller L, Stevens MC, Sahl R (2010). Abnormal functional connectivity of default mode sub-networks in autism spectrum disorder patients. NeuroImage..

[137] Monk CS, Peltier SJ, Wiggins JL, Weng SJ, Carrasco M, Risi S (2009). Abnormalities of intrinsic functional connectivity in autism spectrum disorders. NeuroImage..

[138] von dem Hagen EAH, Stoyanova RS, Baron-Cohen S, Calder AJ (2013). Reduced functional connectivity within and between ‘social’ resting state networks in autism spectrum conditions. Soc Cogn Affect Neurosci.

