# Correction: EEG resting-state functional connectivity: evidence for an imbalance of external/internal information integration in autism

**DOI:** 10.1186/s11689-022-09464-8

**Published:** 2022-10-16

**Authors:** Prany Wantzen, Patrice Clochon, Franck Doidy, Fabrice Wallois, Mahdi Mahmoudzadeh, Pierre Desaunay, Christian Mille, Jean-Marc Guilé, Fabian Guénolé, Francis Eustache, Jean-Marc Baleyte, Bérengère Guillery-Girard

**Affiliations:** 1 Normandie univ, UNICAEN, PSL Université Paris, EPHE, INSERM, U1077, CHU de Caen, GIP Cyceron, Neuropsychologie et Imagerie de la Mémoire Humaine, 14000 Caen, France; 2 Université de Paris, LaPsyDÉ, CNRS, F-75005 Paris, France; 3 INSERM UMR-S 1105, GRAMFC, Université de Picardie-Jules Verne, CHU Sud, 80025 Amiens, France; 4 Centre Ressources Autisme Picardie, Service de Psychopathologie Enfants et Adolescents, CHU, 4 rue Grenier et Bernard, 80000 Amiens, France; 5 Service de Psychiatrie de l’enfant et de l’adolescent, Centre Hospitalier Interuniversitaire de Créteil, 94000 Créteil, France

**Correction: J Neurodevelop Disord 14, 47 (2022)**

10.1186/s11689-022-09456-8

Following publication of the original article [1], the authors identified error in the author names. The given names and family names were erroneously transposed.

The incorrect author name is:

Wantzen Prany, Clochon Patrice, Doidy Franck, Wallois Fabrice, Mahmoudzadeh Mahdi, Desaunay Pierre, Mille Christian, Guilé Jean-Marc, Guénolé Fabian, Eustache Francis, Baleyte Jean-Marc and Guillery-Girard Bérengère*

The correct author name is: Prany Wantzen, Patrice Clochon, Franck Doidy, Fabrice Wallois, Mahdi Mahmoudzadeh, Pierre Desaunay, Christian Mille, Jean-Marc Guilé, Fabian Guénolé, Francis Eustache, Jean-Marc Baleyte and Bérengère Guillery-Girard*

The author group has been updated above and the original article [1] has been corrected.
